# Dataset on interactions of membrane active agents with lipid bilayers

**DOI:** 10.1016/j.dib.2020.105138

**Published:** 2020-01-16

**Authors:** Md Ashrafuzzaman, C.-Y. Tseng, J.A. Tuszynski

**Affiliations:** a Department of Biochemistry, College of Science, King Saud University, Riyadh, 11451, Saudi Arabia; b Department of Oncology, University of Alberta, Edmonton, Canada; c Department of Physics, University of Alberta, Edmonton, Canada; d DIMEAS, Politecnico di Torino, Corso Duca degli Abruzzi, 24, Torino, TO, 10129, Italy

**Keywords:** Drugs, Lipid membrane, Ion channel, Direct detection method, Electrostatic interactions

## Abstract

We address drug interactions with lipids using *in silico* simulations and *in vitro* experiments. The data article provides extended explanations on molecular mechanisms behind membrane action of membrane-active agents (MAAs): antimicrobial peptides and chemotherapy drugs. Complete interpretation of the data is found in the associated original article ‘charge-based interactions of antimicrobial peptides and general drugs with lipid bilayers’ [1]. Data on molecular dynamic simulations of the drug lipid complexes are provided. Additional data and information are provided here to explain the connectivity among various information and techniques used for understanding of the membrane action and/or binding of MAAs including aptamers. Brief explanation has been provided on the possibility of achieving a converted triangle from newly discovered quadrangle, sides of which explain four different phenomena: ‘membrane effects’, ‘detection and quantification’, ‘origin of energetics’ and ‘structure stability’ while drug effects occur. Triangle or quadrangle corners represent various techniques that were applied.

**Table:** Specifications Table

Subject	Biophysics
Specific subject area	Physics behind membrane adsorption of drugs
Type of data	Graph Figure Drawing
How data were acquired	*In silico* molecular dynamic simulations *In vitro* binding Modeling
Data format	Raw Analyzed Drawing/modeling
Parameters for data collection	In silico parameterization on membrane ingredients in *in vitro* aqueous phase and molecular dynamic simulation set up
Description of data collection	Using *in silico* molecular dynamic simulations we collected most of the data. The *in silico* parameterization was made based on parameters utilized *in vitro* experiments in aqueous phase. The structures of lipids and drugs have been modeled considering them as single molecules. For details, see the attached ‘supplementary Data’
Data source location	Institution: King Saud University City/Town/Region: Riyadh Country: Saudi Arabia and Institution: Alberta University City/Town/Region: Edmonton Country: Canada
Data accessibility	See the link: https://data.mendeley.com/datasets/t8n3yk8rvv/1 https://doi.org/10.17632/t8n3yk8rvv.1 Data are stored in the following facilities: Pharmamatrix cluster hosted at University of Alberta, Edmonton, Canada And Biophysics laboratory, Biochemistry Department, King Saud University, Riyadh For additional details on the status of raw data, please consult with the attached ‘supplementary Data’ (see the section ‘Data accessibility’)
Related research article	Ashrafuzzaman M, Tseng CY, Tuszynski J (2019). Charge-based interactions of antimicrobial peptides and general drugs with lipid bilayers. Journal of Molecular Graphics and Modeling, in press, https://doi.org/10.1016/j.jmgm.2019.107502

**Table:** 

**Value of the Data** • These are raw data which explain the trend of individual parameters considering the experimental conditions applied in our associated article [1]. • All who wish to reproduce similar data for general understanding of drug effects in membranes using any similar agents will benefit. • These data provide the scientific trends of a few key biological parameters in standard experimental conditions. The parameters will be utilized as background biophysical information in addressing the distribution, diffusion and toxicity of the drugs while being explored in cell line assays. • Some of these data provide possible altered conditions that satisfy the universality of the claims • Some of these data and supplementary materials provide background parameters of a patented technology ‘direct detection method’.

## 1 Data description

### 1.1 MD simulation of gA and Alm-lipid interactions

**Fig. 1:**
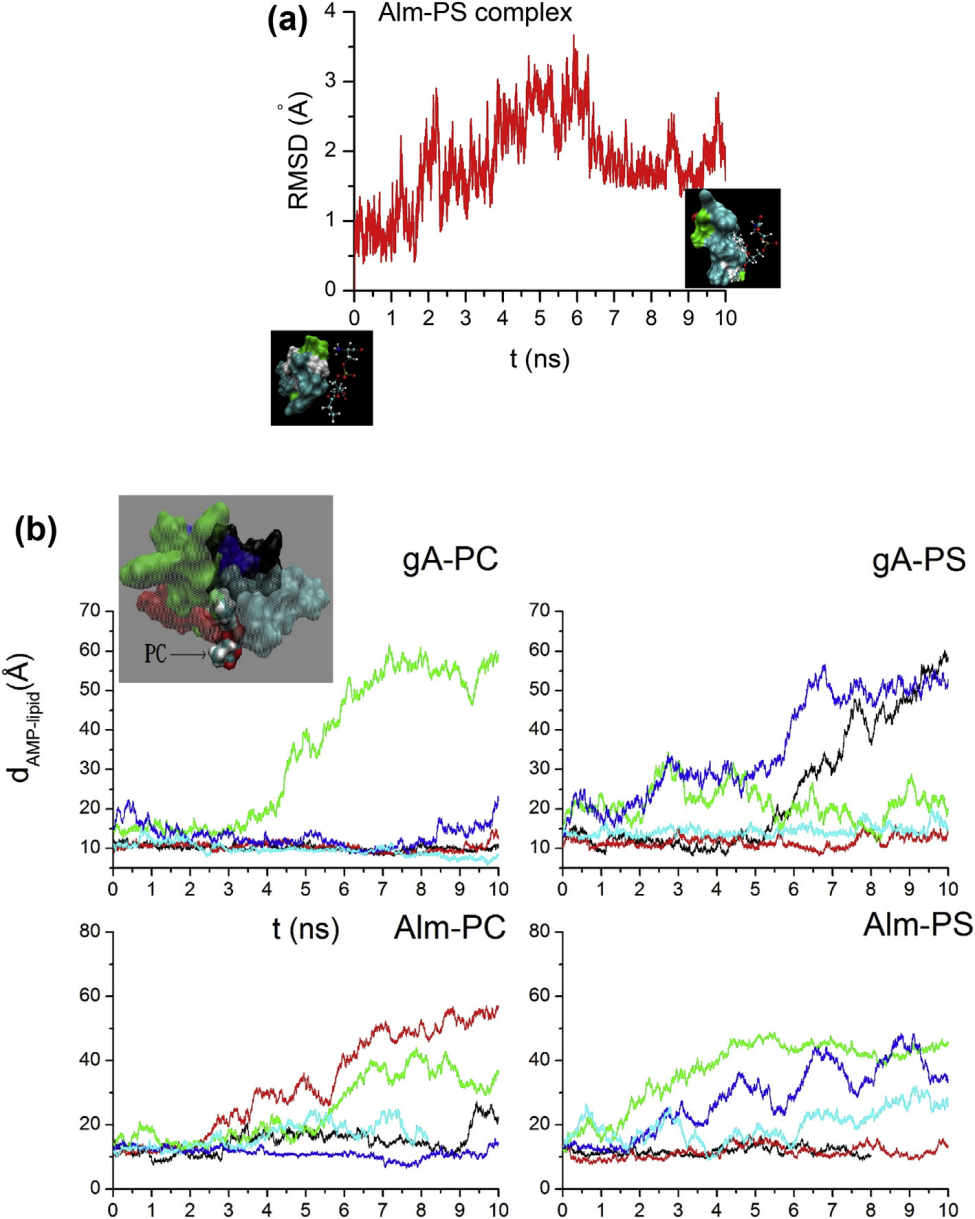
(a) Root mean square deviation of the backbone of Alm in 10 ns simulation (red curve in Figure 1(a)) with initial conformation of Alm-PS shown in the left lower inset. The right inset shows the conformation of Alm-PS at the end of the simulation. (b) Molecular dynamics simulation data representing the change in the AMP–lipid center of mass distance d_AMP_–lipid with time [2]. Five curves with different colors represent five independent initial AMP–lipid complexes. The inset shows the cartoon representations of initial structures of five AMP-lipid complexes with AMP following the color of the corresponding curve.

**Fig. 2:**
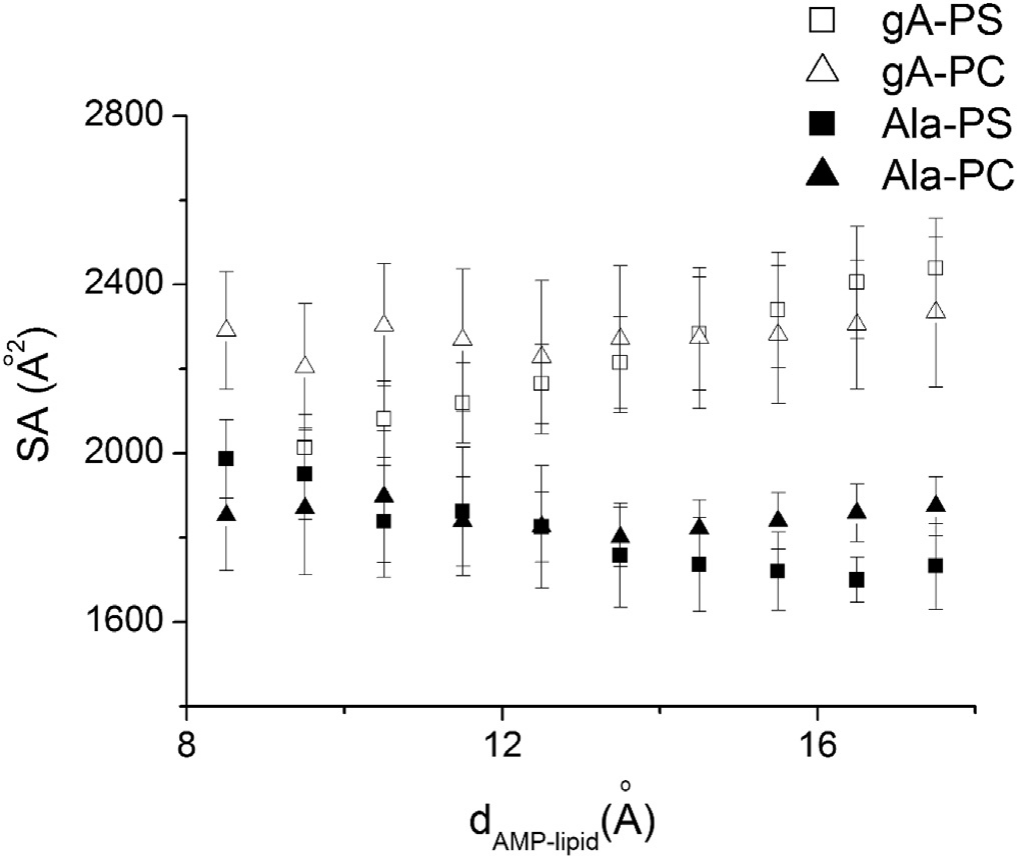
Solvent-accessible (SA) areas for four complexes are plotted against the AMP–lipid center of mass separation distance *d*_*AMP–lipid*_ [2]. It shows SA areas are fluctuating around 2300 and 1800 square angstrom for gA-lipid and Ala-lipid, respectively, which are independent of *d*_*AMP–lipid*_ for the four complexes.

**Fig. 3:**
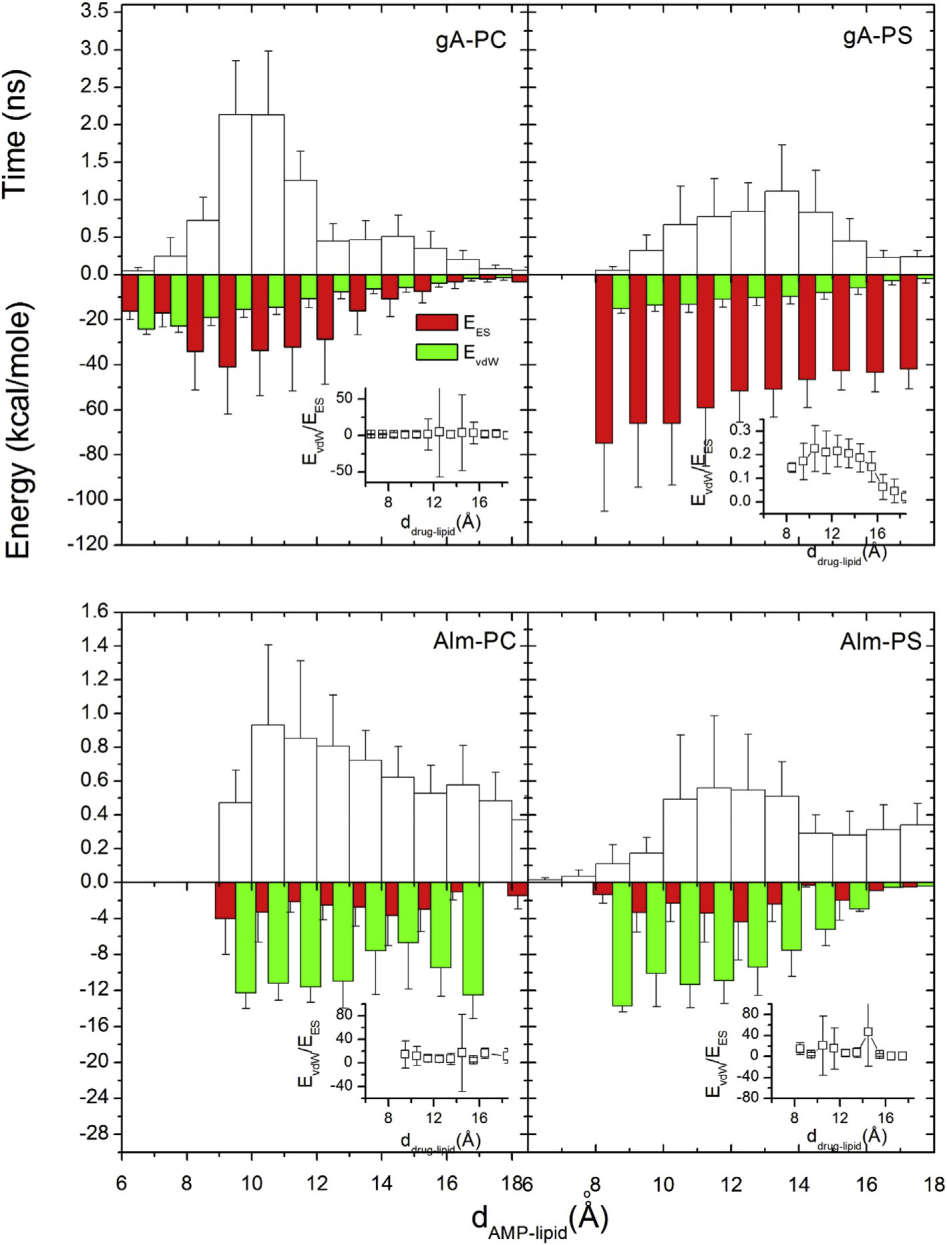
In all four histogram plots (upper panel) of time versus d_AMP–lipid_, the time durations when AMP/lipid stay together (height) within a distance (width) during 10 ns simulations are presented [2]. Lower panels show the histograms of non-bonded van der Waals (vdW) energy (E_vdW_) and electrostatic (ES) interactions energy (E_ES_). To avoid color conflict, E_vdW_ and E_ES_ are shown to occupy half-half widths, although each half represents the whole width of the corresponding histogram.

In this section, we shall present a few figures that represent the summary sketches of simulation data files included in associated link (see doi.org/10.1016/j.dib.2019.104047). The data that were used to generate Fig. 1, Fig. 2, Fig. 3 are uploaded in this repository. Using *in silico* molecular dynamic (MD) simulations we collected these data. For details, see the attached ‘supplementary Data’.

We first examine whether antimicrobial peptide (AMP) tertiary structure is roughly equilibrated under our simulation settings [[2], [3], [4], [5]]. For example, Fig. 1(a) shows the root mean square deviation (RMSD) of the backbone of peptide alamethicin (Alm) in a 10 ns simulation with the initial conformation of Alm-phosphatidylserine (PS) complex that is shown in the lower left inset. The figure shows that RMSD tends to come back down to around 2 Å range after 6 ns. This suggests that the structure of Alm seems to have approached the equilibrium. The conformation of the Alm-PS complex at the end of this simulation is shown in the lower right inset. We turn our attention here to the dynamics of the interactions between AMP and the lipids with consideration given to five different conformations.

In Fig. 1 (b), we present the molecular dynamic (MD) simulation data where we have used five complexes of AMPs and lipids. Here we have plotted the distance of separation between the AMP and lipid molecule centers of mass (*d*_AMP-lipid_) versus the ns order simulation time t. Note that *d*_AMP-lipid_ was used here as the simplest property, yet traditionally used in this kind of trajectory inspection studies, to quantify the AMP-lipid interaction effects. In Fig. 1(b), *d*_AMP-lipid_ is clearly found to fluctuate around 10 Å, similarly to the initial setting in 2–3 simulations in gramicidin A (gA)-phosphatidylcholine (PC), Alm-PC and gA-PS, Alm-PS. In most of these simulations, AMPs and lipids were found to gradually separate.

The solvent accessible (SA) surface area of the complex in the MD simulations was used to investigate whether the hydrophobic effects contribute to AMP-lipid binding. Fig. 2 presents the SA areas in all four cases against *d*_AMP-lipid_. When both AMP and lipid molecules are completely separated we can expect them to be entirely exposed to solvent, i.e., the corresponding SA areas reach the maximum. Fig. 2 shows that the SA areas in all four cases are unchanged between the start and the investigated 18 Å length. This suggests that within this range the drug-lipid complexes stay at an equilibrium solvation condition.

Histograms of *d*_AMP-lipid_ from all five 10 ns simulations and the corresponding energy contributions from two non-bonded interactions, van der Waals (E_vdW_) and electrostatic forces (E_ES_) versus *d*_AMP-lipid_ are shown in Fig. 3. The histogram of *d*_AMP-lipid_ shows that both gA and Alm spent more than 2 ns within 6 Å<*d*_drug-lipid_ <12 Å and away from lipids most of the time (see the upper panels in Fig. 3). It suggests the possibility for AMPs to briefly bind with lipids. Fig. 3 (top panel) indicates that gA likely favors the interaction with PC over PS while Alm shows no significant lipid specific preference. Both E_ES_ and E_vdW_ for gA and Alm interacting with PC are roughly inversely proportional to *d*_AMP-lipid_ while there are weak trends in either gA-PS or Alm-PS cases. It is shown that both E_ES_ and E_vdW_ are strongly effective within 12 Å (E_vdW_ slightly dominant except gA-PS case as shown in the inset plots of Fig. 3). Both E_ES_ and E_vdW_ are larger for gA than for Alm (see the difference in the ranges of energy values along the y-axis in Fig. 3).

### 1.2 Sides of the quadrangle (Fig. 7, original article [1])

There are four sides or lines of a quadrangle and in our case the four sides explain the following four different phenomena: (1) ‘membrane effects’, (2) ‘detection and quantification’, (3) ‘origin of energetics’ and (4) ‘structure stability’. The diagonal line acts to represent the structure-energy connectivity. They are explained here.

### 1.3 DDM↔(EP, FL) is ‘membrane effects’ line

It describes the connection between cause-like membrane active agent (MAA)-lipid binding and effect-like creation of MAA-lipid structures, e.g., ion channels. Peptides, chemotherapy drugs (CDs) and aptamers are generalized as MAAs here.

Experimental technique electrophysiology (EP) indirectly provides information on direct MAA effects on lipid bilayer membranes. Here, the membrane effect creating quantitative measurements on MAA's are performed indirectly from the buffer while it is expected that the membrane concentration of MAAs is different from that in the buffer.

Experimental measurement of fluorescence (FL) provides information based on direct membrane binding but can't help measure quantitative binding with the membrane directly from the membrane. Here, the quantitative measurements on membrane-bound MAA's are performed indirectly through measurements of fluorescence. That means a quantitative response on the binding is measured via intensity of fluorescence corresponding to the MAA's concentration in buffer. No direct detection MAAs in the membrane is possible here. Moreover, the attachment of fluorescence tags with some sites of the MAA molecules changes the chemical and physical properties, which also modulate their membrane-binding propensity.

Direct detection method (DDM) measures the quantitative binding with the membrane directly from the membrane. This makes DDM very unique and it is a method that can provide information on the number of MAA molecules bound to target lipids. Both EP and FL fail to measure the number of molecules of MAAs bound to molecular targets.

### 1.4 DDM↔MD is the ‘detection and quantification’ line and (EP, FL)↔MD is the line for ‘structure-energy connectivity’

These lines provide information on the sources of energies causing MAAs and lipids to interact with each other and thus a fraction of the molecules bind to each other, which causes membrane effects. The MAA-lipid binding due to interactions is quantified using DDM that provides an average binding fraction that is the percentage of MAA molecules bound to lipids. It is also a measure of binding probability. MD estimates major energies in kJ/mole that play roles in the molecular mechanisms to ensure the membrane effects (observed in EP and FL) via MAA binding with lipids. MD thus provides real energy based calculation of binding probability functions. The line connecting DDM and MD is therefore a direct connection between two quantitative measurements of binding probabilities. The link between MD raised information and EP, FL information is indirect and weaker than with DDM information and that is why the line is thinner.

### 1.5 MD↔(Theory, NC) is the ‘origin of energetics’ line

As MD predicts quantitative amounts of binding energies, the theory provides correct analytical expressions that elucidate the origins of those MD measured energies. NC also provides the distance-dependent MAA-lipid interaction energetics and even real numbers on the binding energies as derived using numerical simulations after incorporating average biological values of related parameters. Theory and NC together provides another very important function called the driving force that drives the participating agents (MAAs and lipids) to each other's proximity so that they bind with the energies discovered from MD. This line of the quadrangle is therefore a strong connector between information found on both edges.

### 1.6 EP↔(Theory + NC) is the ‘structure stability’ line

Theory and NC-derived driving force becomes directly correlated with the stability (lifetime) of MAA-lipid binding/interaction raised structures as observed from the *in vitro* experimental data, e.g. ion channels as recorded using EP. This line makes a very strong relationship between information derived from experiments and theory.

### 1.7 Is it possible for the quadrangle to be converted into a triangle?

All the mentioned *in vitro* experimental techniques: DDM, EP and FL altogether interrogate membrane effects of MAAs due to their detectable membrane binding phenomenon. Therefore, if there was a single *in vitro* experimental technique (let's call it ‘super experimental technique (SET)’) that might be able to perform two things, namely to discover the MAA-lipid complex and at the same time to detect the quantitative amount of MAAs bound to lipids, the two edges of the quadrangle covering two different types of *in vitro* studies would merge into one point. The quadrangle then would be converted naturally into a triangle. To the best of our knowledge there is no such technique available yet. Therefore, for the time being our quadrangle appears as a universal diagram illustrating the combination of techniques, which generally address the membrane effects of MAAs or define an agent as a suitable drug for membrane binding.

## 2 Experimental design, materials, and methods

**Fig. 4:**
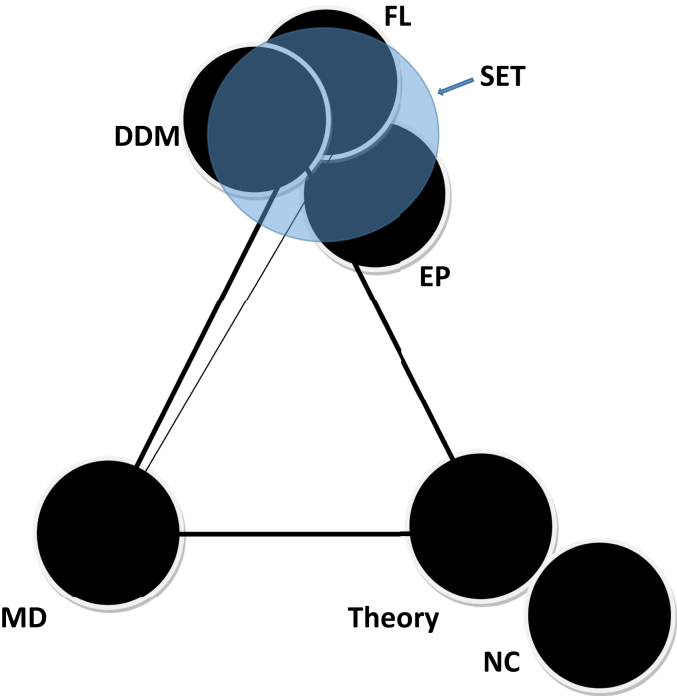
A (virtual) universal triangle connecting various information on MAA-lipid interactions using various techniques. Other techniques may also fit in this triangle to produce similar information.

This Data article is associated to a research article [1], where various methods have been used. These are summarized in Fig. 4 (triangle, this data article) or in Fig 7 (quadrangle, research article [1]). The corners of the triangle represent various techniques. Theory and numerical computation (NC) utilized in research [1] make a corner. They help model complexes created due to long-to-short range MAA-lipid interactions in membranes and compute the free energetics of MAA-lipid association/dissociation using screened Coulomb interactions among distributed charges in the complex. Mathematica 9 was used to program the numerical computations, where experimentally detected values of various parameters have been utilized. MD simulation makes an independent corner. It helps address the *in silico* simulations of the time dependent relative dynamics of the drug and target lipid. These simulations produced data lead to calculating the electrostatic and van der Waals energies among pairs of MAAs and lipids. All the experimental techniques, e.g. DDM, EP, and FL, altogether referred as SET, make another independent corner. SET might produce data to help perform two things, namely to discover the nature of the MAA-lipid complex and at the same time to detect the quantitative amount of MAAs bound to target lipids. DDM specifically helps quantify the agents directly at the binding site. EP, a membrane patch clamp technique, helps measure currents across *in vitro* constructed lipid membranes being doped with specific MAA. Fluorescence measurement technique FL helps indirectly to inspect the drug binding to targets, without providing any measurements on specific mole number for drugs bound to the target structure. DDM serves to quantify agents bound to targets. OriginPro software was used to analyze experimental data and plot.

MAAs used here were chosen from three distinctive classes [1]: Two antimicrobial peptides gramicidin A and alamethicin, two chemotherapy drugs colchicine and taxol, and a set of DNA aptamers that were originally discovered as biomarkers to detect phosphatidylserine externalization in apoptotic cells. Phosphatidylserine and phosphatidylcholine lipids were used for constructing liposomes and planar lipid bilayers. Details are provided in associated original article [1].

The additional descriptions on all the theoretical, computational and experimental methods, information on instruments and design of the experiments, utilized programs and materials, and their compositions in constructed model systems in controlled physiological buffer phases have been briefly provided in the associated research article (see Ref. [1]), and to be detailed in a co-submitted article in another Elsevier journal MethodsX (see Ref. [6]).
