# Supplementary material for: Dataset on interactions of membrane active agents with lipid bilayers
Source: Data Brief. 2020 Jan 16;29:105138. doi: 10.1016/j.dib.2020.105138 (PMC6992954; doi:10.1016/j.dib.2020.105138)
Supplement: Multimedia component 1 [file mmc1.docx]

**Article Title**

Dataset on interactions of membrane active agents with lipid bilayers

**Authors**

Md. Ashrafuzzaman^1,*^, C.-Y. Tseng^2^ and J. A. Tuszynski^2,3,4^

**Affiliations**

^1^Department of Biochemistry, College of Science, King Saud University, Riyadh 11451, Kingdom of Saudi Arabia.

Department of Oncology^2^, Department of Physics^3^, University of Alberta, Edmonton, Canada.

^4^DIMEAS, Politecnico di Torino, Corso Duca degli Abruzzi, 24, Torino, TO, 10129, Italy

**Corresponding author(s)**

^*^ Md. Ashrafuzzaman (mashrafuzzaman@ksu.edu.sa)

**Supplementary Data**

**Description of Data Collection**

Using *in silico* molecular dynamic simulation software Amber 12 we generated molecular trajectory data given structures of lipids and drugs, which have been modelled considering them as single molecules. We then post processed these trajectory data using Amber 12 software package AmberTool, VMD: Visual Molecular Dynamic software and OriginPro: Data Analysis and Graphing software to produce Figure 1-3. The *in silico* parameterization was made based on parameters utilized *in vitro* experiments in aqueous phase and Amber 12 default force fields. Please refer to Journal MethodsX [6] for details of parameterization and simulation set up.

**Data Accessibility**

The original data related to Figs. 1-3 in the Data article are provided in the following link: <https://data.mendeley.com/datasets/t8n3yk8rvv/1>

*Additional information related to data accessibility.* All of the molecular dynamic simulation trajectory results used have been published in various Journals including the associated original article [1-5]. These data have been produced during a decade long active simulation works. They are included in at least 400 trajectory files, stored at the Pharmamatrix cluster hosted at University of Alberta premise. Each of these files has at least 100 gigabytes. Totally, there are at least 4 terabytes (TBs) data. Given settings and parameterization illustrated in [1, 6], same simulation results can be reproduced easily. Uploading all of these trajectories files containing humongous amount of data (4-5 TBs altogether) in public repositories naturally suffers huge issues related to research time ( ̴ weeks-to-month), operating cost, searching, indexing and preservation. Therefore, specific data set (in addition to those presented in above mentioned link) can be accessed upon requests. In the associated original article [1], we have presented binding data for binary combinations of six membrane active agents and two lipids. Fig. 1 and Figs. 3-5 of ref. [1] are plotted using experimental data and these figures are self-explanatory, where either the original data or their mean and standard deviations are plotted. Regarding the simulation data (what are processed in Fig. 6 of associated original article [1] and in Figs. 1-3 in this Data article), we have explained above how we stored the data, besides uploading in the link.
